# Grants to Hospitals Recommended by the Distribution Committee

**Published:** 1917-12-22

**Authors:** 


**KING EDWARD'S HOSPITAL FUND**—(*continued*).

### Grants to Hospitals Recommended by the Distribution Committee.

*The Distribution Committee desire to draw attention to the fact that it must not be assumed that the reduction or absence of a grant implies dissatisfaction.*

**Alexandra Hospital for Children,** £200. **Beckenham Cottage Hospital,** £50, of which £25 to improvements. Belgrave Hospital, £1,000. Blackheath and Charlton Hospital, £100. Bolingbroke Hospital, £750. British Hospital for Mothers and Babies, Woolwich, £200; a grant of £1,500 was set aside in 1913 towards the building of the amalgamated hospital, in accordance with scheme to be approved. **Canning Town Women's Settlement Hospital** (late Medical Mission), £550, of which £50 to reduce debt. Central London Ophthalmic Hospital, £500. Central London Throat and Ear Hospital, capital grants amounting to £10,500 are set aside and promised for the purpose of an amalgamation of the Throat, Nose, and Ear Hospitals (see paragraph 9 of Report). Charing Cross Hospital, £8,000, of which £5,000 to extinguish mortgage debt (see paragraph 7 of Report). Chelsea Hospital for Women, £2,500, of which £2,000 to reduce debt on rebuilding on new site in accordance with the scheme submitted to the Fund. Cheyne Hospital for Sick and Incurable Children, £400, of which £100 to reduce general fund debt. City of London Hospital for Diseases of the Chest, Victoria Park, £5,500, of which £2,000 to reduce deficit on recent improvements in accordance with the scheme submitted to the Fund. City of London Lying-in Hospital, £1,000, of which £250 to reduce general fund debt. Clapham Maternity Hospital, £1,000, of which £400 to reduce debt. **Dreadnought Hospital,** £3,500. **East End Mothers' Lying-in Home,** £600. East London Hospital for Children, £4,500, of which £1,500 is in recognition and aid of the special efforts made to extricate the hospital from serious financial embarrassment (see paragraph 4 of Report). Eltham and Mottingham Cottage Hospital, £100. **Finchley Cottage Hospital,** £100. Florence Nightingale Hospital for Gentlewomen, £300, of which £100 to reduce debt. French Hospital, £200. **General Lying-in Hospital,** £1,350, of which £1,000 to deferred scheme of improvements (see paragraph 5 of Report). German Hospital, £100. Gordon Hospital for Fistula, etc., —. Great Northern Central Hospital, £6,000, of which £2,000 to reduce debt. Grosvenor Hospital for Women, £500. Guy's Hospital, £9,500, of which £2,000 to reduce deficit on recent building improvements in accordance with the scheme submitted to the Fund. **Hampstead General Hospital,** £3,650, of which £150 as agreed upon amalgamation with North-West London Hospital, and £1,500 in recognition and aid of the special efforts made to extricate the hospital from serious financial embarrassment (see paragraph 4 of Report). Hendon Cottage Hospital, £25. Hornsey Cottage Hospital, £50. Hospital and Home for Incurable Children, Hampstead, £50, in consideration of the fact that curable cases are admitted. Hospital and Home for Sick Children, Sydenham, £500, of which £200 to reduce debt. Hospital for Consumption, Brompton (including Sanatorium at Frimley), £5,000. Hospital for Diseases of the Throat, £1,400, of which £900 is maintenance grant, as agreed, upon amalgamation with the London Throat Hospital, and £500 is a grant towards loss incurred by postponement of building scheme through the war. Hospital for Epilepsy and Paralysis, £1,250. Hospital for Sick Children, £5,000, of which £1,000 to reduce debt. Hospital for Women, Soho Square, £2,250, of which £1,000 to reduce debt. Hospital of St. John and St. Elizabeth, £500. **Infants' Hospital,** £500, of which £250 to reduce debt. Italian Hospital, £400. **Kensington and Fulham General Hospital,** £75. Kensington Dispensary and Children's Hospital, £25. King Edward Memorial Hospital (Ealing), £650. King's College Hospital, £7,500, of which £4,500 to reduction of debt on removal to South London, making £67,100 in all contributed by the Fund to this purpose. **Leyton, Walthamstow, and Wanstead Children's and General Hospital,** £550. London Fever Hospital, £200. London Homœopathic Hospital, £1,500, of which £500 to reduce general fund debt. London Hospital, £15,500, of which £1,500 to new nurses' home in accordance with the scheme submitted to the Fund. London Lock Hospital, £3,000, of which £1,500 to maintenance of Harrow Road Hospital, and £1,500 to sanitary and heating improvements

**KING EDWARD'S HOSPITAL FUND**—(*continued*).

at Harrow Road in accordance with the scheme submitted to the Fund. London Temperance Hospital, £3,000, of which £1,250 to reduction of debt on maintenance account. **Maternity Charity and District Nurses' Home, Plaistow,** £400. Memorial Hospital, Mildmay Park, £250. Metropolitan Hospital, £3,000. Middlesex Hospital, £6,000, of which £1,000 to recent improvements. Middlesex Hospital Cancer Charity, £150. Mildmay Mission Hospital, £500. Miller General Hospital for South-East London, £1,600. **National Hospital for Diseases of the Heart,** £200. National Hospital for the Paralysed and Epileptic, £3,500, of which £500 to reduce debt. Nelson Hospital (late South Wimbledon, etc.), £450. New Hospital for Women, £1,000. Norwood Cottage Hospital, £150. **Paddington Green Children's Hospital,** £1,500, of which £700 is in recognition and aid of the special efforts made to extricate the hospital from serious financial embarrassment (see paragraph 4 of Report). Passmore Edwards Acton Hospital, £200. Passmore Edwards East Ham Hospital, £100. Passmore Edwards Hospital for Willesden, £100. Passmore Edwards Cottage Hospital, Wood Green, £50. Poplar Hospital, £300. Prince of Wales's General Hospital, £4,000, of which £1,500 to reduce general fund debt. **Queen Charlotte's Lying-in Hospital,** £2,150, of which £250 to reduce debt, and £650 to deferred scheme of improvements (see paragraph 5 of Report). Queen Mary's Hospital for the East End, incorporating West Ham and Eastern General Hospital, £5,000, of which £1,000 to scheme of improvements to be submitted to the Fund, and £1,000 in recognition and aid of the special efforts made to improve the financial position of the hospital (see paragraphs 4 and 5 of Report). Queen's Hospital for Children, £4,500, of which £1,000 to improvement of out-patient department in accordance with scheme to be submitted to the Fund, and £500 in recognition and aid of the special efforts made to extricate the hospital from serious financial embarrassment (see paragraphs 4 and 5 of Report). **Royal Dental Hospital of London,** £400. Royal Eye Hospital, £600. Royal Free Hospital, £3,000. Royal Hospital for Diseases of the Chest, City Road, £1,250, to reduce debt. Royal Hospital, Richmond, £300. Royal London Ophthalmic Hospital, £2,500. Royal National Orthopædic Hospital, £1,000, of which £250 to reduce building fund debt. Royal Waterloo Hospital for Children and Women, £2,500, of which £1,000 to reduce bank loan. Royal Westminster Ophthalmic Hospital, £150. **St. Andrew's Hospital (Dollis Hill),** £50. St. Columba's Hospital, £250, of which £100 to reduce debt. St. George's Hospital, £4,500. St. John's Hospital for Diseases of the Skin, —. St. John's Hospital, Lewisham, £400. St. Luke's Hospital for Advanced Cases, £150. St. Mark's Hospital, £400. St. Mary's Hospital, £3,500. St. Mary's Hospital for Women and Children, Plaistow, £2,250, of which £500 to urgent temporary improvement in nurses' and servants' quarters and out-patient department in old hospital building, and £1,000 to deferred scheme of rebuilding (see paragraph 5 of Report). St. Monica's Home Hospital, £200, of which £100 to reduce debt. St. Peter's Hospital for Stone, £300. St. Saviour's Hospital, Osnaburgh Street, £200. St. Thomas's Hospital, £1,000. Salvation Army Maternity Hospital, £300. Samaritan Free Hospital, £1,500, of which £500 to reduce debt. Santa Claus Home, £50. Stoke Newington Home Hospital for Women, £150. **University College Hospital,** £5,500, of which £500 to improvements to lifts. **Victoria Hospital for Children,** £1,000. **West End Hospital for Nervous Diseases,** £400. Western Ophthalmic Hospital, £600, of which £500 to reduction of mortgage debt. West London Hospital, £5,000, of which £1,500 to reduction of building fund loan. Westminster Hospital, £5,300, of which £3,500 to maintenance, £1,500 to reduce general fund debt, and £300 annual grant to acquisition of new site as agreed. Wimbledon Hospital, £100. Woolwich and Plumstead Cottage Hospital, £25.

**Total Grants to Hospitals for the Year 1917, £181,000.**

